# 2206. Pragmatic Assessment of Influenza Vaccine Effectiveness in the Department of Defense (PAIVED): Updates from Year 4 of a Multi-site Trial

**DOI:** 10.1093/ofid/ofac492.1825

**Published:** 2022-12-15

**Authors:** Timothy Burgess, Stephanie A Richard, Limone Collins, Christina Spooner, Srihari Seshadri, Christina Schofield, Anuradha Ganesan, Wesley R Campbell, David Hrncir, Tahaniyat Lalani, Tyler Warkentien, Katrin Mende, Ana E Markelz, Catherine M Berjohn, Bruce McClenathan, Jitendrakumar Modi, Alan Williams, Rhonda E Colombo

**Affiliations:** Infectious Disease Clinical Research Program, Department of Preventive Medicine and Biostatistics, Uniformed Services University of the Health Sciences, Bethesda, MD, USA, Bethesda, Maryland; Infectious Disease Clinical Research Program, Department of Preventive Medicine and Biostatistics, Uniformed Services University of the Health Sciences, Bethesda, MD, USA, Bethesda, Maryland; Immunization Healthcare Division, Defense Health Agency, Bethesda, Maryland; Immunization Healthcare Division, Defense Health Agency, Bethesda, Maryland; Immunization Healthcare Division, Defense Health Agency, Bethesda, Maryland; Madigan Army Medical Center Division of Infectious Diseases, Infectious Disease Clinical Research Program, Tacoma, Washington; Infectious Disease Clinical Research Program, Department of Preventive Medicine and Biostatistics, Uniformed Services University of the Health Sciences, Bethesda, MD, USA, Walter Reed National Military Medical Center, Bethesda, Maryland; Walter Reed National Military Medical Center, Bethesda, Maryland; Carl R. Darnall Army Medical Center/Wilford Hall Ambulatory Surgical Center, Fort Hood, Texas; Naval Medical Center Portsmouth, Portsmouth, Virginia; Naval Medical Center Portsmouth, Portsmouth, Virginia; Infectious Disease Clinical Research Program, Department of Preventive Medicine and Biostatistics, Uniformed Services University of the Health Sciences, Bethesda, MD, USA, Bethesda, Maryland; Brooke Army Medical Center, San Antonio, Texas; Naval Medical Center San Diego Division of Infectious Diseases, Infectious Disease Clinical Research Program, San Diego, CA; Womack Army Medical Center, Fort Bragg, North Carolina; Naval Health Clinic, Annapolis, MD, Anapolis, Maryland; Uniformed Services University of the Health Sciences, Bethesda, Maryland; Infectious Disease Clinical Research Program, Department of Preventive Medicine and Biostatistics, Uniformed Services University of the Health Sciences, Bethesda, MD, USA, The Henry M. Jackson Foundation for the Advancement of Military Medicine, Madigan Army Medical Center Division of Infectious Diseases, Tacoma, Washington

## Abstract

**Background:**

The effectiveness of the influenza vaccine is varies with circulating strain concordance and timing of influenza spread in a community. The Pragmatic Assessment of Influenza Vaccine Effectiveness in the DoD (PAIVED) study is a multi-year, randomized clinical trial of three FDA-licensed vaccine types (egg-based, cell-based, and recombinant), designed to determine which influenza vaccine platform is most effective among adults in a military setting.

**Figure 1.**
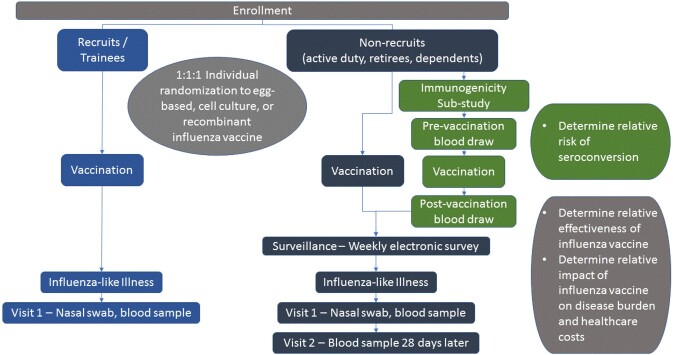
PAIVED summary flow chart

**Methods:**

Participants in the fourth year of PAIVED (2021-22 influenza season) were enrolled from September 2021 through January 2022 at 9 military facilities. Participants were asked each week about influenza-like illness (ILI) symptoms. If the participants reported ILI symptoms, research staff scheduled an acute and convalescent ILI visit. Additional details about the study are included in Figure 1.

**Results:**

In year 4, 4,688 participants were enrolled, among whom 63.8% were male, 56.5% were white, and the average age was 34 years (Tables 1 and 2). As of early April, 1,297 ILIs had been reported. Most participants reported a single ILI (987 (87%)), while 140 participants reported two ILIs and 10 reported three ILIs. The mean duration of the reported ILIs was 11 days, with a mean 5 days of limited activity. Three participants were hospitalized. Among the samples processed to date, influenza has been identified in four participants. The most common pathogens in year 4 were SARS-CoV-2 and rhino/enterovirus (Figure 2). During all four years of PAIVED, we enrolled 15,449 participants, among whom 188 episodes of influenza have been identified so far (1.2%).

**Table 1.**
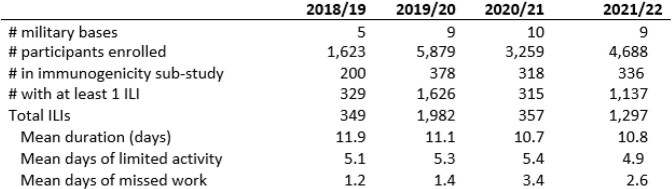
PAIVED summary over four seasons

**Table 2.**
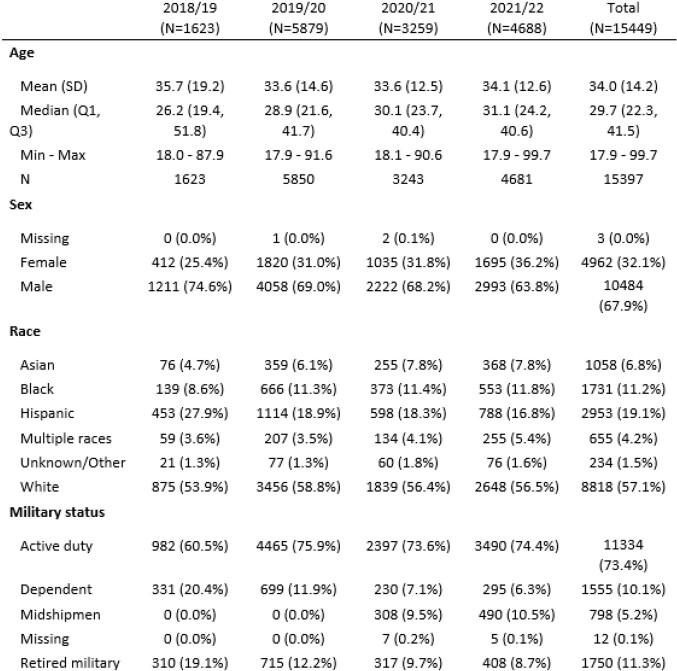
Demographic characteristics of PAIVED participants during four seasons

**Figure 2.**
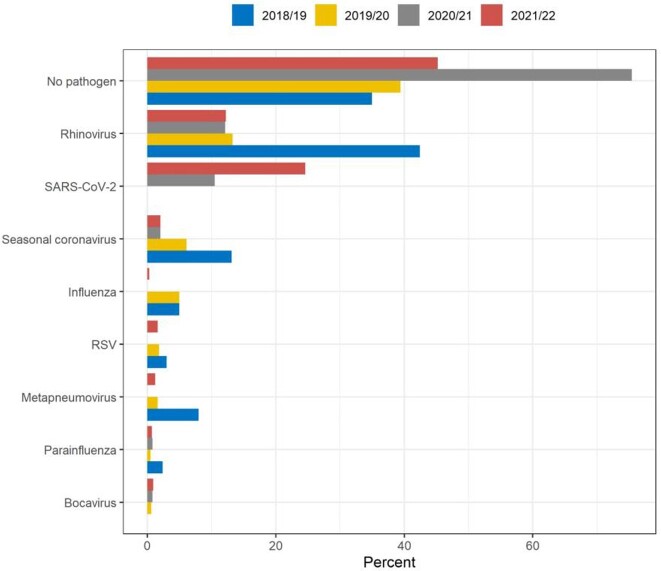
Pathogens identified in ILI swabs collected in PAIVED (2021/22 season still in progress)

**Conclusion:**

The fourth year of PAIVED was characterized by early (pre-enrollment) spread of influenza in some areas, as well the nationwide spread of the SARS-CoV-2 Omicron variant in December. As the swabs are processed and participants’ military health records are reviewed, we expect to identify more influenza cases; however, transmission patterns were far lower than historical averages due to pandemic precautions, making this surveillance data from identified strains more valuable. Comparative influenza vaccine effectiveness calculations will be performed to inform future vaccine purchasing decisions and we will compare serological response to the different vaccines.

**Figure:**
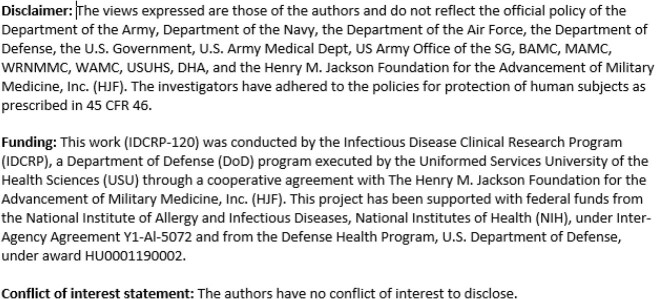

**Disclosures:**

**Timothy Burgess, MD, MPH**, AstraZeneca: The HJF, in support of the USU IDCRP, was funded to conduct or augment unrelated Phase III Mab and vaccine trials as part of US Govt. COVID19 response **Jitendrakumar Modi, MD**, GlaxoSmithKline: I am a paid speaker for GSK. I do not speak for their flu brand.

